# Ursolic Acid Alleviates Mitotic Catastrophe in Podocyte by Inhibiting Autophagic P62 Accumulation in Diabetic Nephropathy

**DOI:** 10.7150/ijbs.94096

**Published:** 2024-06-11

**Authors:** Hang Mei, Tienan Jing, Haojun Liu, Yue Liu, Xinwang Zhu, Jiao Wang, Li Xu

**Affiliations:** 1 Department of Laboratory Medicine, The Second Affiliated Hospital of Guangdong Medical University, Zhanjiang, Guangdong 524003, PR China.; 2 Department of Nephrology, The First Hospital of China Medical University, Shenyang, 110001, PR China.; 3 Department of Oral Mucosa Disease, School and Hospital of Stomatology, China Medical University, Liaoning Provincial Key Laboratory of Oral Diseases, Shenyang, 110002, PR China.; 4 Department of Orthodontics, The Second Affiliated Hospital of Guangdong Medical University, Zhanjiang, Guangdong 524003, PR China.; 5 Department of Obstetrics and Gynecology, Shengjing Hospital of China Medical University, Shenyang, 110004, PR China.

**Keywords:** Diabetic nephropathy (DN), Ursolic acid (UA), podocyte, P62, autophagy, mitotic catastrophe

## Abstract

The glomerular podocyte, a terminally differentiated cell, is crucial for the integrity of the glomerular filtration barrier. The re-entry of podocytes into the mitotic phase results in injuries or death, known as mitotic catastrophe (MC), which significantly contributes to the progression of diabetic nephropathy (DN). Furthermore, P62-mediated autophagic flux has been shown to regulate DN-induced podocyte injury. Although previous studies, including ours, have demonstrated that ursolic acid (UA) mitigates podocyte injury by enhancing autophagy under high glucose conditions, the protective functions and potential regulatory mechanisms of UA against DN have not been fully elucidated. For aiming to investigate the regulatory mechanism of podocyte injuries in DN progression, and the protective function of UA treatment against DN progression, we utilized db/db mice and high glucose (HG)-induced podocyte models in vivo and in vitro, with or without UA administration. Our findings indicate that UA treatment reduced DN progression by improving biochemical indices. P62 accumulation led to Murine Double Minute gene 2 (MDM2)-regulated MC in podocytes during DN, which was ameliorated by UA through enhanced P62-mediated autophagy. Additionally, the overexpression of NF-κB p65 or TNF-α abolished the protective effects of UA both *in vivo* and *in vitro*. Overall, our results provide strong evidence that UA could be a potential therapeutic agent for DN, regulated by inhibiting podocyte MC through the NF-κB/MDM2/Notch1 pathway by targeting autophagic-P62 accumulation.

## 1. Introduction

The latest epidemiological data from the International Diabetes Federation (IDF) indicates that 537 million individuals globally suffer from diabetes mellitus (DM), a number projected to rise to approximately 783 million by 2045 1. Diabetic nephropathy (DN), as a chronic microvascular complication of DM, is the primary cause of end-stage renal disease (ESRD), posing a significant threat to public health 2. DN is characterized by proteinuria, overproduction of the mesangial matrix, renal hypertrophy, and fibrosis 3. Although the strict control of blood glucose control can delay the progression of DN, few interventions can halt or reverse kidney dysfunction 4. Consequently, elucidating the underlying mechanisms of DN progression is crucial to identifying potential therapeutic targets of great significance.

The glomerular podocyte, a highly differentiated specialized visceral cell, is located on the outer surface of the glomerular basement membrane (GBM) and, together with the perforated endothelial cell, constitutes the glomerular filtration barrier (GFB). Injured podocytes undergo foot process effacement and detachment, exposing denuded areas on the glomerular capillaries, which results in proteinuria 5. Moreover, the loss of podocytes, whether through death or detachment, precedes the increased volume of glomeruli, providing strong evidence for early-stage DN diagnosis 6. Under physiological conditions, mature podocytes are meant to be quiescent cells arrested in the G0 (resting) phase. However, podocytes may inappropriately enter the cell cycle and activate mitosis by losing cell-cycle checkpoints, a process known as " mitotic catastrophe (MC)", triggered by chemical or physical stresses that leads to abnormal chromosome segregation. As a result, cell morphology, especially the structure of foot processes, cannot be maintained during the mitotic process due to the lack of cytoskeletal proteins, leading to podocyte detachment from the GBM and/or cell death 7, 8. Murine Double Minute gene 2 (MDM2) is expressed in glomeruli and plays a role in podocyte cell cycle regulation 7, 9. However, the signaling pathways related to MDM2-regulated MC in DN remain unclear.

It is generally accepted that cells exhibit multiple stress or metabolic reactions to maintain homeostasis. Among these, autophagy serves as a critical self-protection mechanism that specifically degrades long-lived or damaged organelles through the formation of mitophagosomes. The autophagic progress typically includes the emergence of a phagophore, autophagosome formation, and fusion with a lysosome to degrade encapsulated substrates, a process known as “autophagic flux” 10. P62 is identified as an autophagy substrate that acts as a receptor for autophagic activity, responsible for delivering ubiquitinated proteins to the proteasome for degradation 11. Thus, impaired autophagic degradation is characterized by the accumulation of autophagy substrates, leading to P62 buildup. Emerging evidence suggests that impaired autophagic flux contributes to podocyte injury and the progression of DN 12, 13. However, the role of P62-mediated autophagic flux in inducing MC in podocytes during DN progression warrants further investigation.

**Figure 1:**
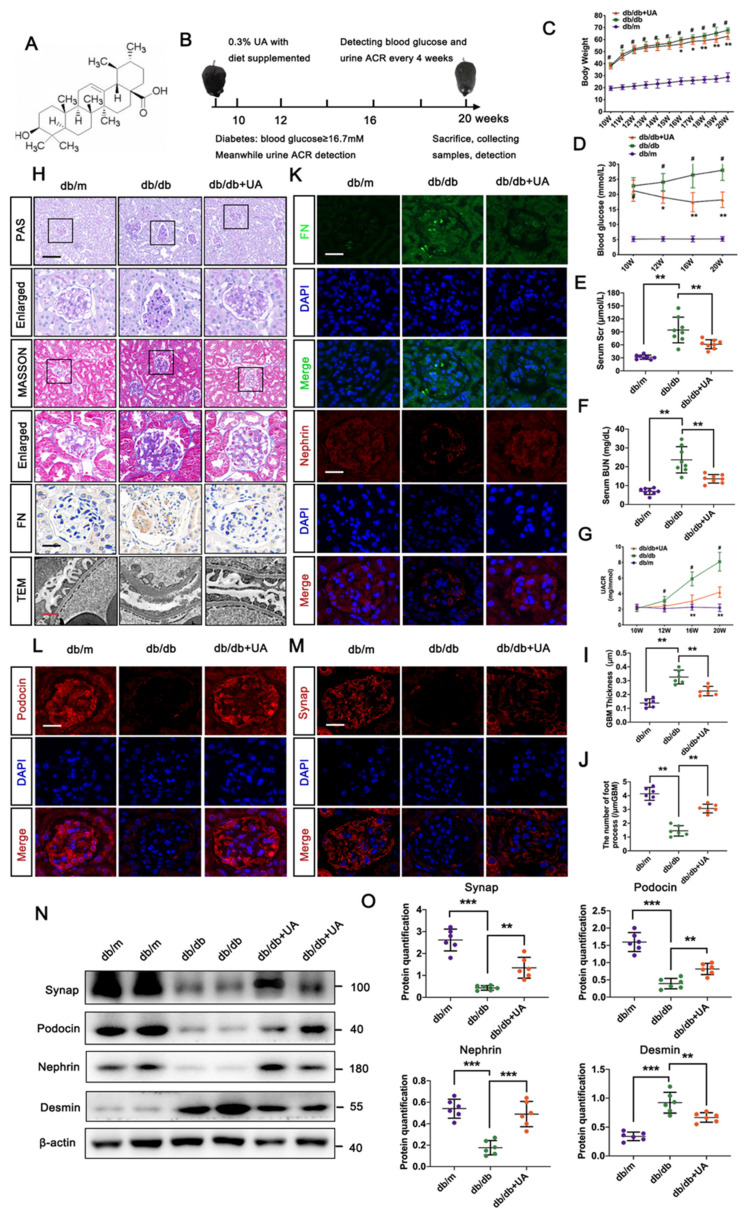
** UA mitigates pathological dysfunction of kidney and podocyte injury in db/db group.** (A) Chemical structure of UA; (B) Schematic illustration of UA administration in the db/db group; (C, D) Body weight and blood glucose levels in db/m, db/db, and db/db + UA group (n=8); (E, F) Levels of serum creatinine (Scr) and blood urea nitrogen (BUN) in db/m, db/db, and db/db + UA groups (n=8) at 20 weeks; (G) Urinary albumin creatinine ratio (UACR) in db/m, db/db, and db/db + UA groups (n=8) at 20 weeks; (H) Representative images of PAS and Masson staining (scale bar=50 μm), TEM observation (scale bars=2 μm), and IHC staining (FN level) (scale bar=20 μm) in db/m, db/db, and db/db + UA groups; (I) Immunostaining for FN (green) and Nephrin (red), counterstained with DAPI (blue) by IF staining in db/m, db/db, and db/db + UA groups (scale bars=20 μm); (J, K) Immunostaining for Podocin (red) and Synap (red), counterstained with DAPI (blue) by IF staining in db/m, db/db, and db/db + UA groups (scale bars=20 μm); (L, M) Protein levels of Synap, Podocin, Nephrin, and Desmin in db/m, db/db, and db/db + UA groups by Western blot, with semi-quantitative analyses (n=6). Data represent the mean ± SD from three independent experiments. ^#^*P*<0.05 versus db/m, and *P<.05, **P<.01 or ***P<.001 versus db/db group by one-way or two-way ANOVA.

Ursolic acid (UA), a pentacyclic triterpene derived from a wide variety of vegetables (Figure 1A), possesses a broad range of pharmaceutical properties and therapeutic effects, including anti-tumor, anti-inflammatory, and anti-proliferative actions 14-16. It has been reported that UA lowers blood glucose concentrations, reduces lipid accumulation, and decreases insulin resistance in diabetic models 17. Similarly, we have previously reported that UA mitigates podocyte injury by enhancing autophagy under high glucose conditions 13. However, the specific autophagic-related mechanisms by which UA aids in podocyte recovery in DN progression have not yet been described.

In the current study, we demonstrate that impaired autophagic flux contributes to MC in DN-related podocyte injury, both *in vivo* and *in vitro*. Furthermore, we present the novel finding that UA administration inhibits the re-entry of cell cycles in podocytes, thus preventing MC via the P62-mediated autophagic pathway in DN. Therefore, exploring the interplay between autophagy flux and MC may be beneficial in elucidating the underlying mechanisms of UA's protective effects against DN progression.

## 2. Materials and Methods

### 2.1 Animal experimental design

The db/db mice and age-matched lean littermate mice (db/m) (BKS.Cg-Dock7m^+/+^ Leprdb/J) were procured from the Model Animal Research Center of Nanjing University. All animal experimental procedures received approval from the Ethics Committee on the Care and Use of Laboratory Animals of China Medical University, in compliance with China's Animal Welfare Legislation. All animals were housed in a standard environment with a regular light/dark cycle under specific pathogen-free (SPF) conditions. Based on prior research 18, the UA-treated db/db group (db/db + UA) was fed a diet containing 0.3% UA (0.3 g of UA per 100 g of standard feed) for 10 weeks, as depicted in Figure 1B. The db/m group served as the control group.

An adeno-associated virus (AAV)-packaged P62 targeted at the specific promoter (Nephrin) of podocyte, referred to as NPHS-P62 OE, were obtained from GeneChem Company (Shanghai, China), and was *in-situ* injected into the renal pelvis of the UA-treated db/db group. Four groups were established as follow: 1. db/m group; 2. db/db group; 3. UA-treated db/db group; 4. UA-treated-db/db group with NPHS-P62 transfection.

### 2.2 Biochemical analysis

At 8 weeks, blood was collected from the tail vein of each group for biochemical analysis. A blood glucose level >16.7mM indicated successful establishment of the diabetic mouse model. Blood glucose, urine creatinine, and urine albumin levels were monitored every 4 weeks as previously described 19. Starting from 10 weeks, UA or AAV-NPHS-P62 OE were administrated, and all groups were fasted for at least 8 h for blood glucose and urine tests (during which water access was permitted). Body weight was measured weekly. Blood and tissue samples were collected post-euthanasia at 20 weeks.

### 2.3 Cell culture and treatment

Conditionally immortalized murine podocytes were obtained from ATCC /Shanghai Cell Bank (BLUEFBIO, number BFN60808809) and cultured as previously described 13, 20. After 14-days culture, podocytes were considered differentiated, as determined by the expression of synaptopodin (Synap). Additionally, podocytes were exposed to 35 mmol/L glucose for 48 h, forming the high glucose (HG) group.

Bafilomycin A1 (Baf A1, 200 nM) were administered 30 min prior to HG exposure. Cultured podocytes were divided into three groups: 1. Normal glucose (NG, 11.1 mmol/L glucose); 2. HG (35 mmol/L glucose); 3. HG + Baf A1 (35 mmol/L glucose + 200 nM Baf A1).

Overexpressed P62 (P62 OE, Syngentech, China) was prepared by amplifying and subcloning into the eukaryotic expression vector pcDNA3.1. Rapamycin (Rapa, Sigma -Aldrich), a classical autophagy agonist, was used in HG-induced podocytes. The podocytes were categorized into four groups: 1. Normal glucose (NG, 11.1 mmol/L glucose); 2. HG (35 mmol/L glucose); 3. HG + Rapa (35 mmol/L glucose+100 nmol/L Rapa); 4. HG + Rapa + P62 OE (35 mmol/L glucose + 100 nmol/L Rapa + P62 OE).

Similarly, UA-treated podocytes under HG conditions were classified into four groups: 1. NG (11.1 mmol/L glucose); 2. HG (35 mmol/L glucose); 3. HG + UA (35 mmol/L glucose+5 μmol/L UA); 4. HG+UA+P62 OE (35 mmol/L glucose + 5 μmol/L UA + P62 OE).

The specific targeting sequence of P62 siRNA (siP62, Syngentech, China) was transfected using Lipofectamine 3000 (Invitrogen) according to the manufacturer's instructions. siP62 and/or TNF-α/overexpressed NF-κB p65 vector, which activates the NF-κB pathway, were introduced in HG-induced podocytes with or without UA treatment. For varying experimental purposes, cultured podocytes were organized into the following groups: 1. HG (35 mmol/L glucose); 2. HG + siP62 (35 mmol/L glucose + siP62); 3. HG + siP62 + TNF-α (35 mmol/L glucose + siP62 + 40 ng/mL TNF-α). Or 1. HG (35 mmol/L glucose); 2. HG + UA (35 mmol/L glucose+5 μmol/L UA); 3. HG + UA + TNF-α (35 mmol/L glucose + 5 μmoL/L UA + 40 ng/mL TNF-α).

### 2.4 Enzyme-linked immunosorbent assay (ELISA)

ELISA was used to measure serum creatinine (Cr, ml037726) and blood urea nitrogen (BUN, ml076479) levels, following the manufacturer's instructions.

### 2.5 Histological and immunohistochemical (IHC) staining

Mouse kidney tissues were sectioned into 3 µm thick slices. For histological analysis, samples underwent Periodic acid Schiff (PAS) and Masson staining. IHC staining was performed according to the manufacturer's protocol. Antibodies specific for FN (Abcam ab2413), p-H3 (Cell Signaling Technology, #9701), Aurora B (Sigma-Aldrich, ZRB1149), Ki-67 (Cell Signaling Technology, #34330), P21 (Proteintech, 28248-1-AP), NF-κB p65 (Abcam, ab16502), MDM2 (Santa Cruz Biotechnology, sc-965), NICD (Abcam, ab52627), and Hes1 (Abcam, ab71559) were incubated overnight at 4 °C, followed by secondary antibodies for 1 h at room temperature. Sections were stained with diaminobenzidine, counterstained with hematoxylin, and examined under a microscope.

### 2.6 Transmission electron microscopy (TEM)

Serial sections (80 nm) of kidney and podocyte samples were prepared using an ultramicrotome and stained with uranyl acetate and lead citrate. After rinsing and vacuum-drying at room temperature, the ultrastructure, particularly the foot process effacement and the glomerular basement membrane (GBM), was examined using TEM 21.

### 2.7 Immunofluorescent (IF) staining

Frozen sections (3 µm) of kidney tissues were fixed with 4% paraformaldehyde for 15 min at room temperature. Podocytes on coverslips were fixed with cold methanol/acetone for 10 min at room temperature. After blocking with 10% donkey serum for 60 min, slides were immunostained overnight at 4 °C with primary antibodies FN (Abcam, ab2413), Nephrin (Santa Cruz Biotechnology, sc-376522), Podocin (Sigma-Aldrich, SAB4200810), Synaptopodin (Sigma-Aldrich, SAB3500585), LC3 (Abcam, ab192890), P62 (Novus, H00008878-M01), α-tubulin (Abcam, ab7291), Ki-67 (Cell Signaling Technology, #34330), Cyclin B1 (Abcam, ab181593), MDM2 (Santa Cruz Biotechnology, sc-965), and Hes1 (Abcam, ab71559). This was followed by a 2-h incubation with secondary antibodies. Cell nuclei were counterstained with DAPI (Sigma-Aldrich) for 10 min. Images were captured using a confocal microscope.

### 2.8 Western blot

Western blot analysis was conducted as previously described 13. The antibodies utilized included: Nephrin (Santa Cruz Biotechnology, sc-376522), Podocin (Abcam, ab181143), Synaptopodin (Sigma-Aldrich, SAB3500585), Desmin (Proteintech, 22205-1-AP), LC3 (Abcam, ab192890), P62 (Abcam, ab109012), p-H3 (Cell Signaling Technology, #9701), Aurora B (Sigma-Aldrich, ZRB1149), Cyclin E (Proteintech, 11554-1-AP), Cyclin D1 (Abcam, ab16663), PCNA (Abcam, ab92552), Cyclin B1 (Abcam, ab181593), P21 (Proteintech, 10355-1-AP for human, 28248-1-AP for mouse), NF-κB p65 (Abcam, ab16502), MDM2 (Santa Cruz Biotechnology, sc-965), NICD (Abcam, ab52627), and Hes1 (Abcam, ab71559).

### 2.9 Flow cytometry

For cell cycle progression, cells were fixed with 70% cold ethanol and stained with propidium iodide (PI) staining solution containing RNase A (10 mg/mL). The podocytes were then incubated at 37°C and analyzed using a FACScan flow cytometer.

### 2.10 Statistical analysis

All quantitative data were derived from independent experiments with triplicate repeats and expressed as mean ± SD. Statistical analyses were performed using a two-tailed unpaired Student's t-test for comparisons between two groups, one-way ANOVA, and the Bonferroni test for multiple comparisons, utilizing SPSS 15.0 software. *P* < 0.05 was considered statistically significant.

## 3. Results

### 3.1 UA prevented DN progression in db/db group

Physiological indices including body weight, blood glucose level, serum creatinine, and urea nitrogen level in the db/db group with or without UA administration are shown in Figure 1C-F. Since initiating UA supplementation at 10 weeks, body weights in the UA-treated group persistently decreased compared to the db/db group, with significant differences observed at 16 weeks. UA administration significantly reduced the blood glucose level in the db/db group starting from 12 weeks. Similar trends in serum creatinine and urea nitrogen levels were observed in both the db/db + UA and db/db groups at 20 weeks. Additionally, UA administration notably reversed the abnormal elevation of the urine albumin/creatinine ratio (UACR) in the db/db group starting from 16 weeks (Figure 1G).

Histological analyses using PAS and Masson staining showed that UA administration alleviated mesangial matrix expansion and mitigated glomerulosclerosis in the db/db group (Figure 1H). Regarding renal ultrastructure, podocyte foot process effacement and thickening of the basement membrane in the db/db group were markedly reversed by UA treatment (Figure 1H). Additionally, the db/db + UA group exhibited a significantly weaker expression of FN in glomeruli compared to the db/db group, indicating a reduced degree of renal fibrosis under diabetic conditions (Figure 1H, I). Consistent with these findings, damage to podocytes was assessed by the expression of podocyte-related markers, including nephrin, podocin, and synaptopodin (Synap). As demonstrated in Figure 1I-M, both immunofluorescent (IF) staining and Western blot analysis confirmed that damaged podocytes in the db/db group were repaired by upregulating the levels of Synap, podocin, and nephrin, and reducing Desmin levels with UA treatment.

### 3.2 UA enhanced autophagy level of DN in db/db group

**Figure 2:**
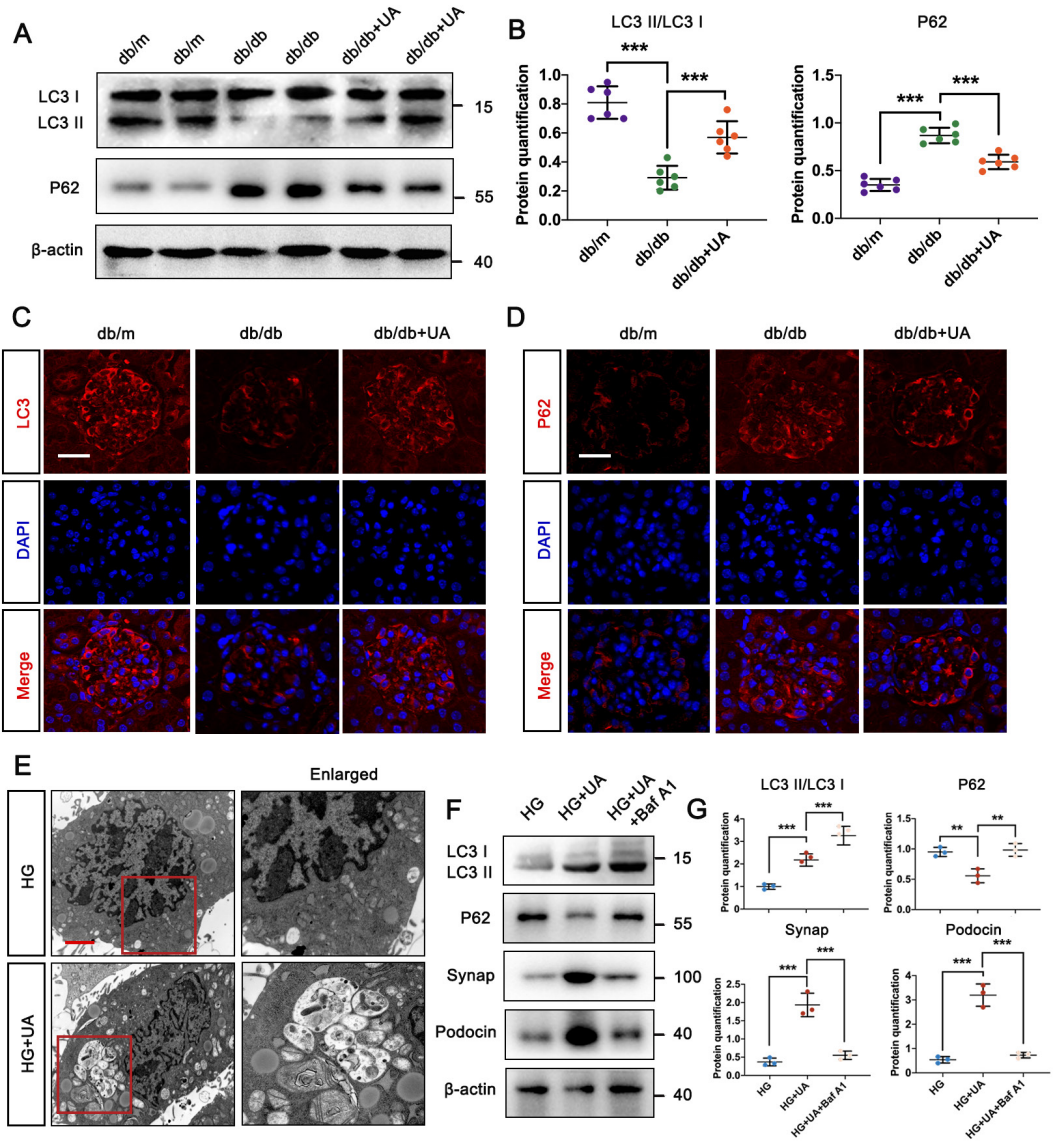
** UA enhances autophagy level of DN in db/db group.** (A, B) Protein levels of LC3II/LC3I and P62 in db/m, db/db, and db/db + UA groups by western blot and their semi-quantitative analyses (n=6); (C, D) Immunostained for LC3 (red) and P62 (red), counterstained with DAPI (blue) by IF staining in db/m, db/db, and db/db + UA groups (scale bar=20 μm); (E) Representative images of autophagic vacuoles in podocytes between NG and HG groups by TEM (scale bar=2 μm); (F, G) Protein levels of LC3II/LC3I, P62, Synap, and Podocin in HG, HG + UA, and HG + UA + Baf A1 groups by western blot and their semi-quantitative analyses (n=3). Data represent the mean ± SD from three independent experiments. **P<.01 or ***P<.001 versus db/db group or HG group by one-way ANOVA.

We previously confirmed that UA attenuated podocyte injury by increasing autophagy under high glucose conditions *in vitro* 13, we first investigated the autophagy levels in the db/db group with or without UA treatment. Western blot and IF staining showed that the ratio of LC3II/LC3I was down-regulated, while the level of P62 was up-regulated in the db/db group (Figure 2A-D), indicating a decrease in overall autophagy under DN conditions. Conversely, UA administration resulted in a significantly higher level of autophagy compared to the db/db group (Figure 2A-D).

To accurately monitor podocyte autophagy, the number of autophagic vacuoles was examined in HG-cultured podocytes *in vitro* using TEM. In Figure 2E, autophagic vacuoles accumulated in the HG + UA group, while few were observed in the HG group. Furthermore, we used Bafilomycin A1 (Baf A1), a typical lysosomal inhibitor, to investigate the direct correlation between UA and autophagy. The impairment of autophagy in the HG + UA group with Baf A1 was verified by the up-regulation of LC3II/LC3I and P62, indicating a failure in autophagosome degradation that led to P62 accumulation. Consistent with these results, the significantly higher levels of Podocin and Synap in the HG + UA group dramatically decreased in the presence of Baf A1, nearly matching the levels in the HG group (Figure 2F, G). Thus, it was demonstrated that the therapeutic effect of UA on podocytes was attributed to an autophagy-dependent manner.

### 3.3 Autophagic P62 accumulation induced podocyte MC in high glucose condition

**Figure 3:**
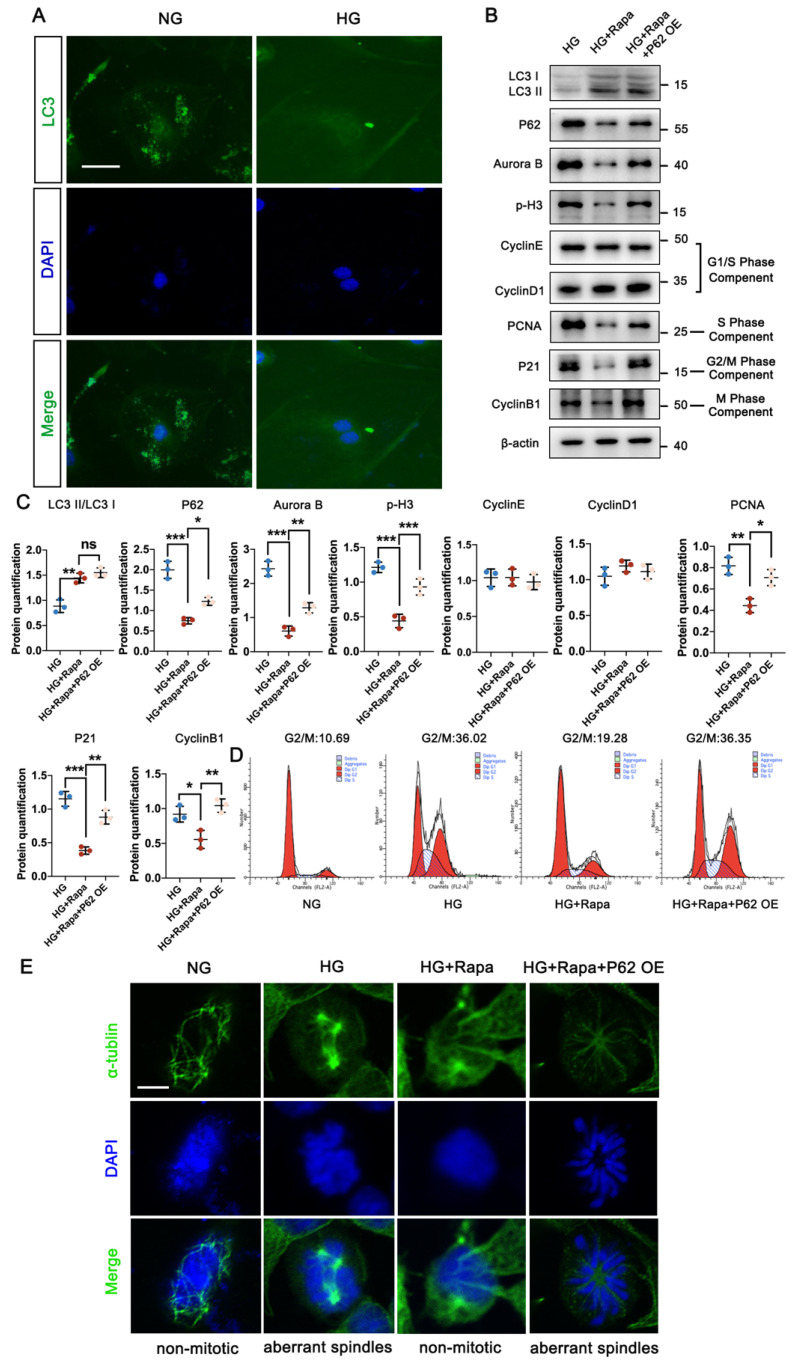
** HG stimulates cell cycle re-entry of podocyte through autophagic P62 accumulation.** (A) Immunostained for LC3 (green), counterstained with DAPI (blue) by IF staining in NG and HG groups (scale bar=10 μm); (B, C) Protein levels of LC3II/LC3I, P62, Aurora B, p-H3, Cyclin E, Cyclin D1, PCNA, P21, and Cyclin B1 in HG, HG + Rapa, and HG + Rapa + P62 OE groups by western blot and their semi-quantitative analyses (n=3); (D) Cell-cycle events in NG, HG, HG + Rapa, and HG + Rapa + P62 OE groups by flow cytometry analysis (n=3); (E) Immunostained for α-tubulin (green), counterstained with DAPI (blue) by IF staining in NG, HG, HG + Rapa, and HG + Rapa + P62 OE groups (n=3). Data represent the mean ± SD from three independent experiments. *P<.05, **P<.01 or ***P<.001 versus HG + Rapa group by one-way ANOVA.

In physiological conditions, mature podocytes are in the quiescent stage (G0 phase), a prerequisite for their highly specialized functions, while an abnormal cell cycle may contribute to podocyte injury in DN progression 22, 23. Under HG conditions, podocytes exhibited significantly lower fluorescent intensity of LC3, accompanied by the abnormal nuclear shapes, compared with the NG group (Figure 3A). Specific immunostaining of α-tubulin suggested HG triggered the formation of abnormal mitotic spindles and aberrant nuclear shapes (Figure 3E). Higher levels of Aurora B and phospho-Histone H3 (p-H3) indicated that podocytes re-entered the cell cycle under HG conditions, confirmed by varying levels of cell-cycle proteins including PCNA, P21, and Cyclin B1, which are checkpoints of the S, G2/M, and M phases. No differences in Cyclin E and Cyclin D1 (G1/S phase checkpoint) were found between the NG and HG groups (Figure S1C). Western blot results showed that the protein expression of P62 in podocytes increased under high glucose conditions, and the expression of Aurora B and p-H3, related to cell MC, also increased. Additionally, Baf A1 intervention further aggravated podocyte MC (Figure S2A, B). Flow cytometry analysis showed a large number of podocytes in the G0/G1 phase entered the S and G2/M phases under HG conditions (Figure 3D). Moreover, Rapamycin (Rapa), a classic autophagic activator, was applied to investigate the potential correlation between MC and autophagy. Rapa enhanced the autophagy process in podocytes by increasing the LC3II/LC3I level and reducing P62 accumulation. The down-regulation of Aurora B and p-H3 suggested the inhibition of the mitotic process treated by Rapa. Furthermore, we assessed the checkpoints of the cell cycle in the presence of Rapa. The expression of PCNA, P21, and Cyclin B1 were up-regulated in podocytes treated with Rapa, while no differences in Cyclin E and Cyclin D1 were found with or without Rapa under HG conditions (Figure 3B, C). Flow cytometry analysis showed that Rapa inhibited podocytes from re-entering the HG-induced G2/M phase, which were supposed to stay at the G0/G1 phase (Figure 3D). Besides, podocytes treated with Rapa lacked mitotic spindles, presenting a non-mitotic state (Figure 3E). Thus, it was demonstrated that activation of autophagy avoided HG-induced abnormal mitotic status in podocytes. However, over-expression of P62 facilitated cell cycle re-entry and suppressed the protective effect of Rapa against HG impairment in podocytes.

### 3.4 P62 accumulation weakened the therapeutic effect of UA on DN in db/db group

**Figure 4:**
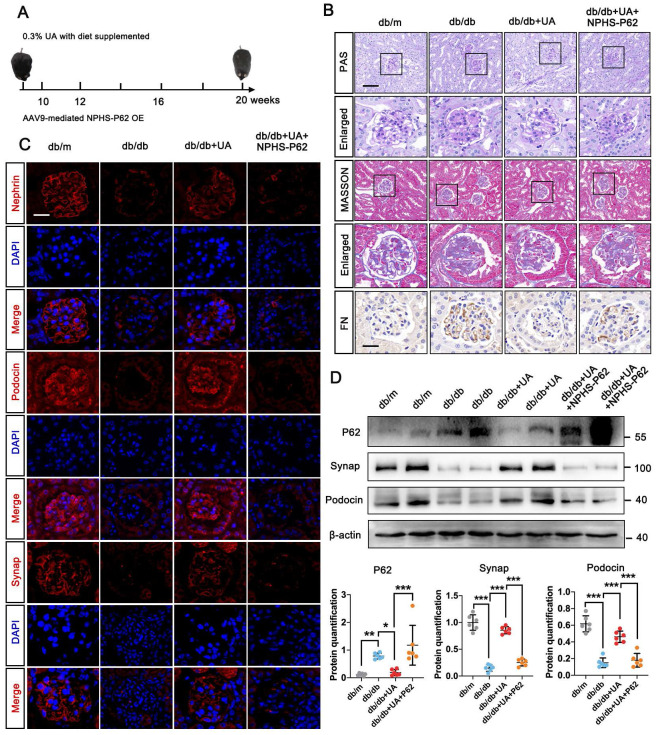
** P62 accumulation weakened the therapeutic effect of UA in db/db group.** (A) Schematic illustration of UA administration and/or AAV9-NPHS-P62 OE injection in the db/db group; (B) Representative images of PAS and Masson staining (scale bars=50 μm), IHC staining for FN level (scale bars=20 μm) in db/m, db/db, db/db + UA, and db/db + UA + NPHS-P62 groups; (C) Immunostained for Nephrin, Podocin, and Synap (red), counterstained with DAPI (blue) by IF staining in db/m, db/db, db/db + UA, and db/db + UA + NPHS-P62 groups (scale bar=20 μm); (D) Protein levels of Synap, Podocin, and Nephrin in db/m, db/db, db/db + UA, and db/db + UA + NPHS-P62 groups by western blot and their semi-quantitative analyses (n=6). Data represented the mean ± SD of three independent experiments. *P<.05, **P<.01 or ***P<.001 versus db/db group or db/db + UA group by one-way ANOVA.

We next evaluated the impact of autophagic P62 accumulation under UA treatment in the db/db group. An over-expressed db/db group was created via in-situ injection of NPHS-P62 OE (Figure 4A). PAS staining revealed mesangial proliferation, matrix accumulation, and capillary stenosis in the glomerular area of the db/db + UA + NPHS-P62 OE group compared with the db/db + UA group (Figure 4B). Additionally, exacerbated collagen deposition and inflammatory cell infiltration were observed in the UA-treated db/db group with NPHS-P62 OE, as shown by Masson staining (Figure 4B). The amelioration of FN deposition under UA treatment was negated by NPHS-P62 OE (Figure 4B). Western blot and IF staining confirmed the reversal of Nephrin, Podocin, and Synap effects in the UA-treated db/db group upon introducing NPHS-P62 OE (Figure 4C and 4D). Thus, it was suggested that P62 accumulation weakened the therapeutic effect of UA in the db/db group.

### 3.5 Over-expressed P62 abolished the protective function of UA on mitotic catastrophe of podocyte under HG conditions

**Figure 5:**
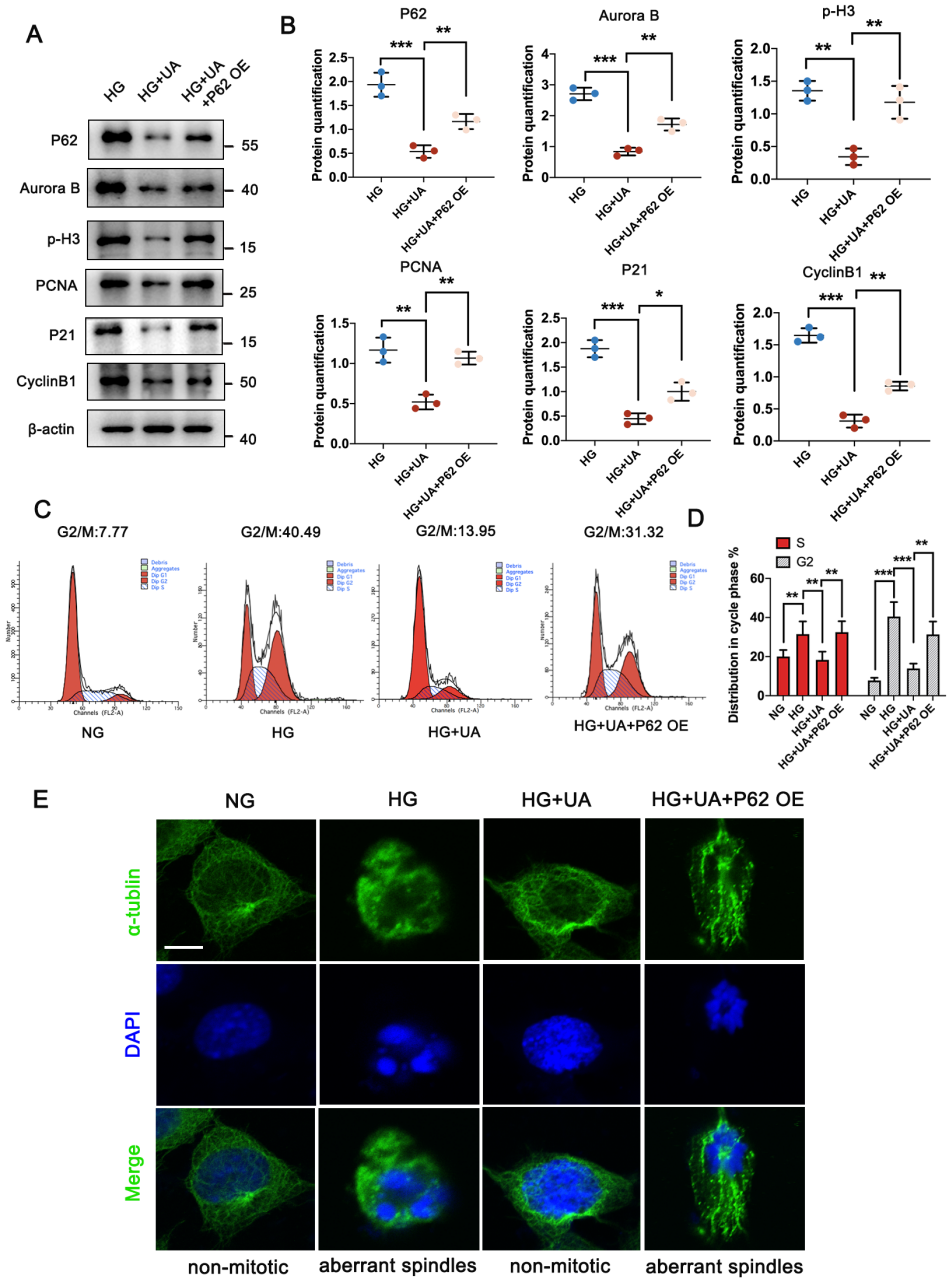
** UA mitigates** MC **in podocytes through P62 regulation under HG conditions.** (A, B) Protein levels of P62, Aurora B, p-H3, PCNA, P21, and Cyclin B1 in HG, HG + UA, and HG + UA + P62 OE groups by western blot, with semi-quantitative analyses (n=3); (C, D) Cell-cycle events of podocytes in NG, HG, HG + UA, and HG + UA + P62 OE groups analyzed by flow cytometry, with semi-quantitative results (n=3); (E) Immunostained for α-tubulin (green) and counterstained with DAPI (blue) in NG, HG, HG + UA, and HG + UA + P62 OE groups by IF staining (n=3). Data represented the mean ± SD of three independent experiments. *P<.05, **P<.01 or ***P<.001 versus HG or HG + UA group by one-way ANOVA.

Considering that enhanced autophagy levels could prevent podocyte MC under HG conditions, it was important to determine whether P62 accumulation could lead to abnormal cell cycles in HG-induced podocytes under UA treatment. As demonstrated in Figure 5A and 5B, UA treatment with over-expressed P62 showed significantly higher levels of Aurora B and p-H3, along with up-regulation of PCNA, P21, and Cyclin B1, compared to the UA + HG group. Flow cytometry analysis revealed an increased ratio of S and G2/M phases in podocytes under HG conditions decreased with UA treatment but recovered with over-expressed P62 (Figure 5C, D). Additionally, over-expressed P62 led to aberrant spindles compared to UA administration under HG conditions (Figure 5E).

**Figure 6:**
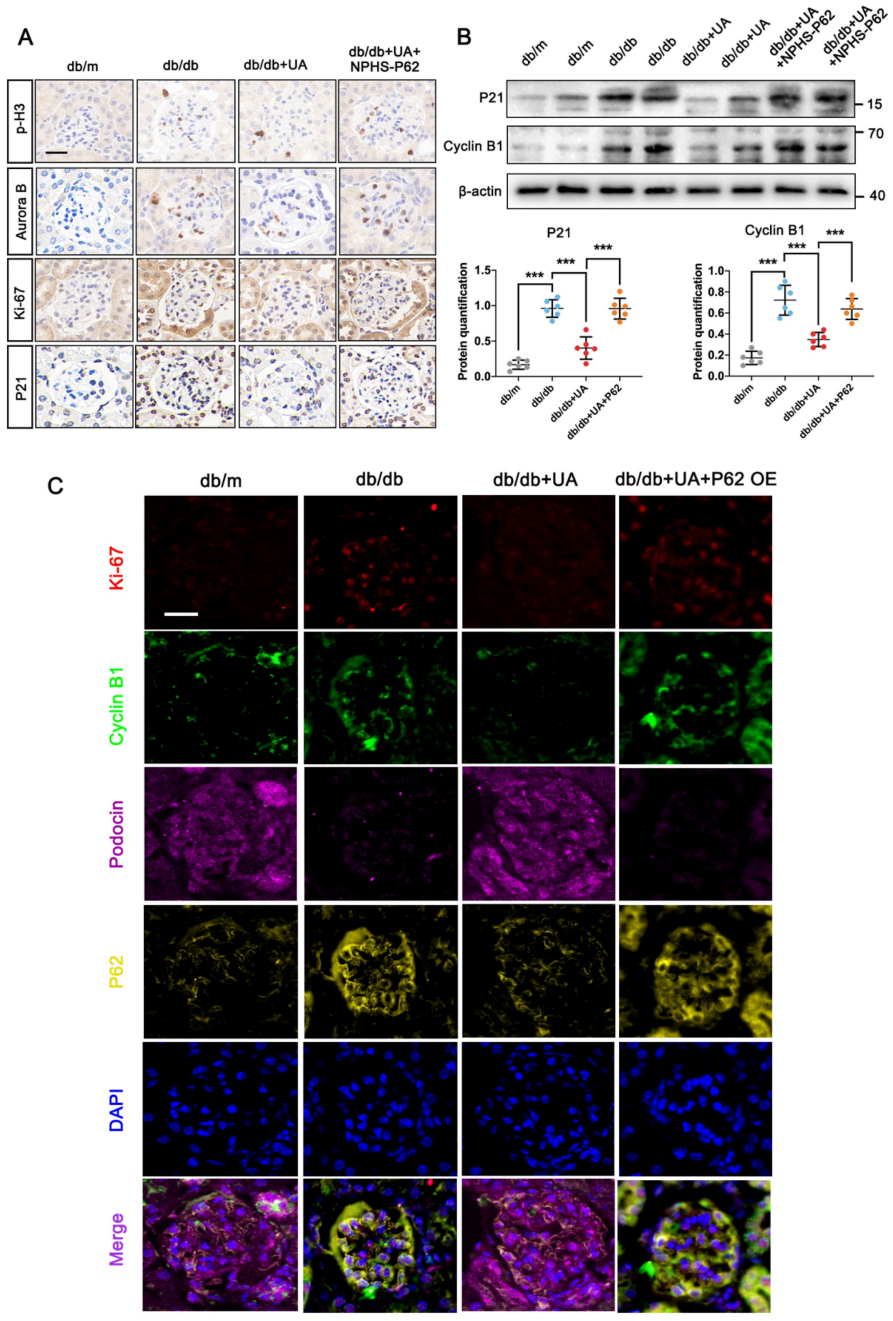
** P62 accumulation diminishes the protective effect of UA on podocyte** MC** in db/db group.** (A) Protein levels of p-H3, Aurora B, Ki-67, and P21 in db/m, db/db, db/db + UA, and db/db + UA + NPHS-P62 groups by IHC assay, scale bar=20 μm; (B) Protein levels of P21 and Aurora B in db/m, db/db, db/db + UA, and db/db + UA + NPHS-P62 group by western blot, with semi-quantitative analyses (n=6); (C) Immunostained for Ki-67 (red) and Cyclin B1 (green), Podocin (pink), and P62 (yellow), counterstained with DAPI (blue) in db/m, db/db, db/db + UA, and db/db + UA + NPHS-P62 groups (scale bar=20 μm). Data represented the mean ± SD from three independent experiments. ***P<.001 versus db/db or db/db + UA group by one-way ANOVA.

Furthermore, the UA-treated db/db group, with or without *in-situ* injection of NPHS-P62 OE, was prepared. The increased levels of p-H3, Aurora B, and Ki-67 in the db/db group were significantly reduced under UA treatment but returned to similar levels in the NPHS-P62 group (Figure 6A). Compared with the db/m group, the down-regulation of P21 and Cyclin B1 in the db/db group suggested podocytes re-entered the cell cycle under diabetic conditions. Although UA administration could maintain podocytes in a quiescent state by reducing the levels of P21 and Cyclin B1, Over-expressed NPHS-P62 weakened the therapeutic effect of UA, resulting in MC of podocytes. Similar trends were observed in quadruple staining of Ki-67 (Red), Cyclin B1 (Green), Podocin (Pink), and P62 (Yellow), indicating glomeruli in the UA-treated group exhibited significantly higher fluorescent intensities of Podocin. The levels of Ki-67, Cyclin B1, and P62 were down-regulated, which were inhibited by over-expressed NPHS-P62. Taken together, UA attenuated podocyte MC through a P62-dependent pathway.

### 3.6 P62-NF-κB-MDM2 axis was involved in MC of podocyte under HG condition

**Figure 7:**
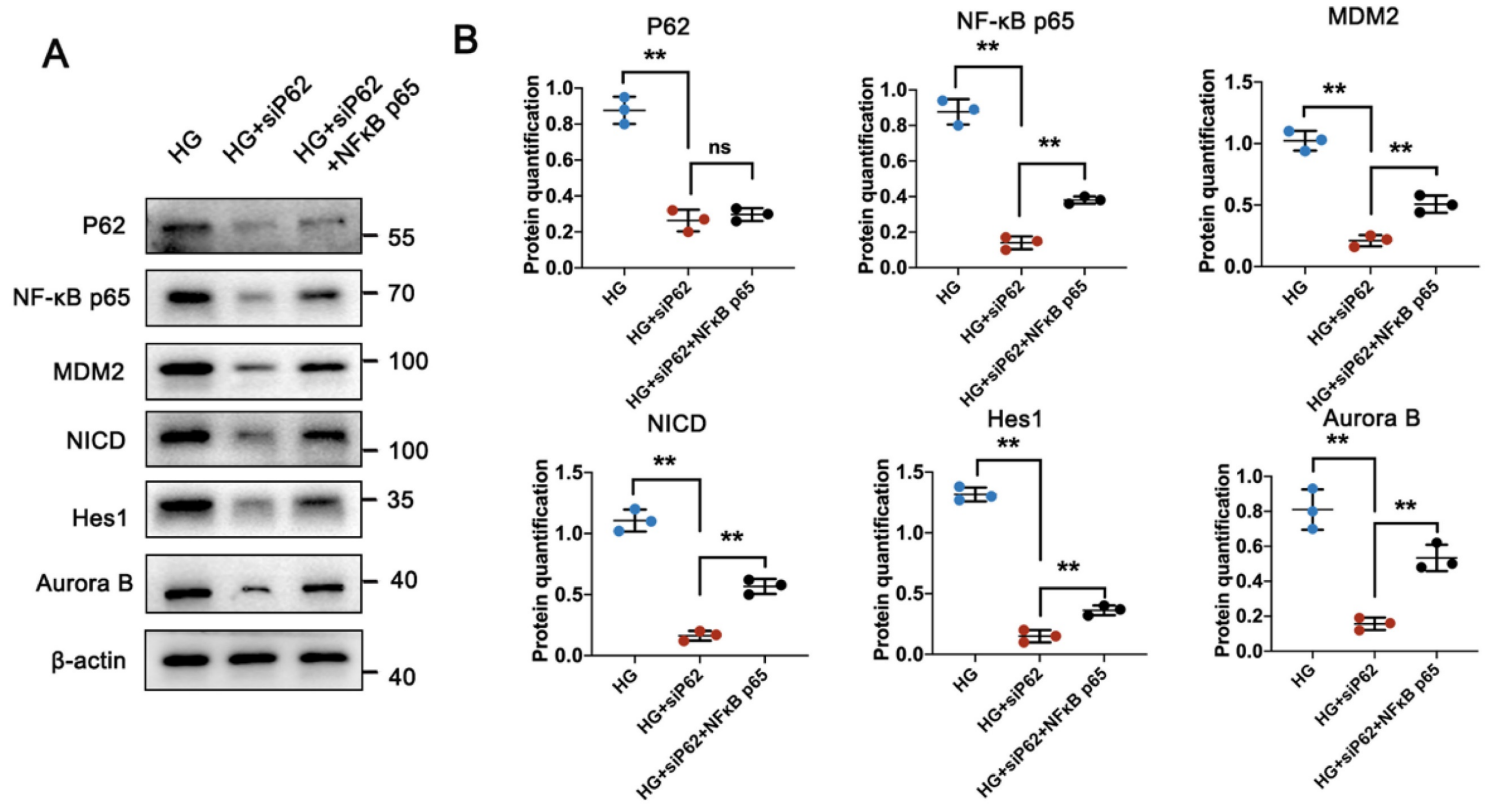
** HG induces podocyte** MC** through P62-mediated NF-κB/MDM2 activation.** (A, B) Protein levels of P62, NF-κB p65, MDM2, NICD, Hes1, and Aurora B in HG, HG + siP62, and HG + siP62 + NF-κB groups by western blot, with semi-quantitative analyses (n=3). Data represented the mean ± SD from three independent experiments. **P<.01 or ***P<.001 versus HG + siP62 group by one-way ANOVA.

P62 contains multiple structural domains that interact with various signaling proteins. It is known that P62 is involved in the tumor necrosis factor α (TNFα)-induced NF-κB signaling pathway, activating its targeted gene, MDM2 24, 25. Consistent with previous studies 22, 26, MDM2 was highly expressed in glomeruli under HG conditions but showed reduced expression under UA treatment. Specifically, the MDM2-downstream Notch1 pathway, responsible for cell differentiation during organism development, involves the Notch intracellular domain (NICD) in DN conditions, which can translocate into the nucleus and upregulate the expression of hairy and enhancer of split 1 (Hes1), leading to podocyte apoptosis in the glomerulus 27, 28. Having confirmed varying expressions of MDM2 in the db/db and db/db + UA groups, we investigated the correlation between P62-mediated MDM2 activation and podocyte MC under HG conditions using a P62 knockout plasmid (siP62) and/or TNF-α, an agonist of the NF-κB pathway. siP62 significantly reduced the expression of NF-κB p65, accompanied by lower expression of MDM2, NICD, and Hes1 (Figure 7A, B and Figure S3A, B). Concurrently, a reduced cell proliferation rate was observed with the downregulation of Aurora B under HG conditions. The introduction of TNF-α or over-expressed NF-κB did not alter P62 levels between the NG and HG groups. In contrast, levels of NF-κB p65 were significantly higher, as well as increased expressions of MDM2, NICD, and Hes1, suggesting that high glucose induces P62-mediated MC of podocytes through the upregulated NF-κB-MDM2 signaling pathway.

### 3.7 UA ameliorated MC of podocyte in DN through the P62-NF-κB-MDM2-Notch1 pathway

**Figure 8:**
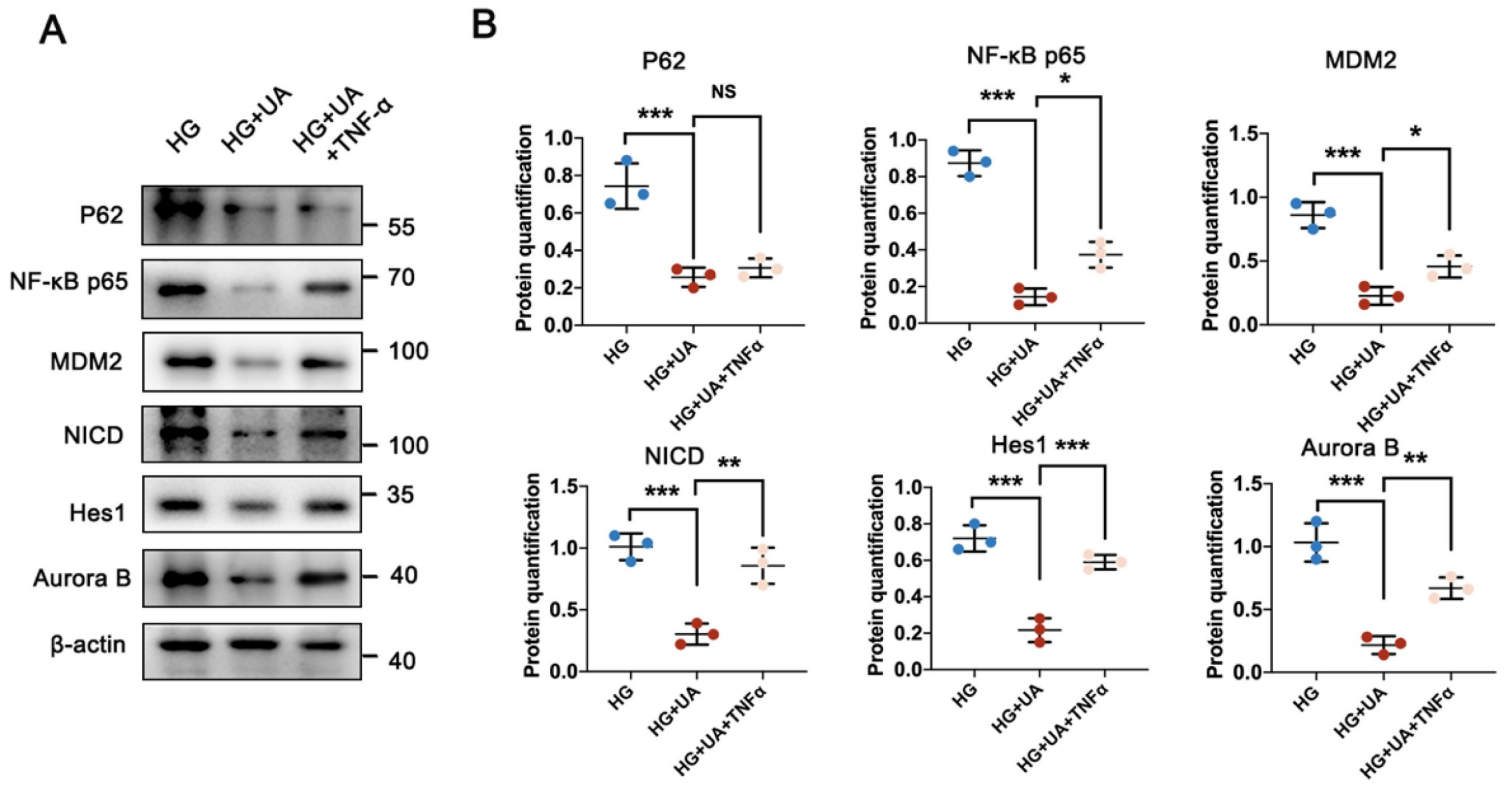
**UA alleviates** MC** in podocytes through P62-mediated NF-κB-MDM2-Notch1 pathway under HG conditions.** (A, B) Protein levels of P62, NF-κB p65, MDM2, NICD, Hes1, and Aurora B in HG, HG + UAm and HG + UA + TNF-α groups by western blot, with semi-quantitative analyses (n=3). Data represented the mean ± SD of three independent experiments. *P<0.05, **P<.01 or ***P<.001 versus HG+UA group by one-way ANOVA.

To elucidate the underlying mechanism of ameliorated MC in podocytes under HG conditions with UA treatment, podocytes were prepared with UA and/or TNF-α under HG conditions. Compared with the HG group, podocyte proliferation in the HG + UA group was suppressed due to the significantly lower P62 level and inhibited activity of the NF-κB-MDM2 pathway, while the addition of TNF-α in the HG + UA group affected the NF-κB-MDM2 pathway, rather than the P62 level, resulting in abnormal podocyte proliferation (Figure 8A, B).

**Figure 9:**
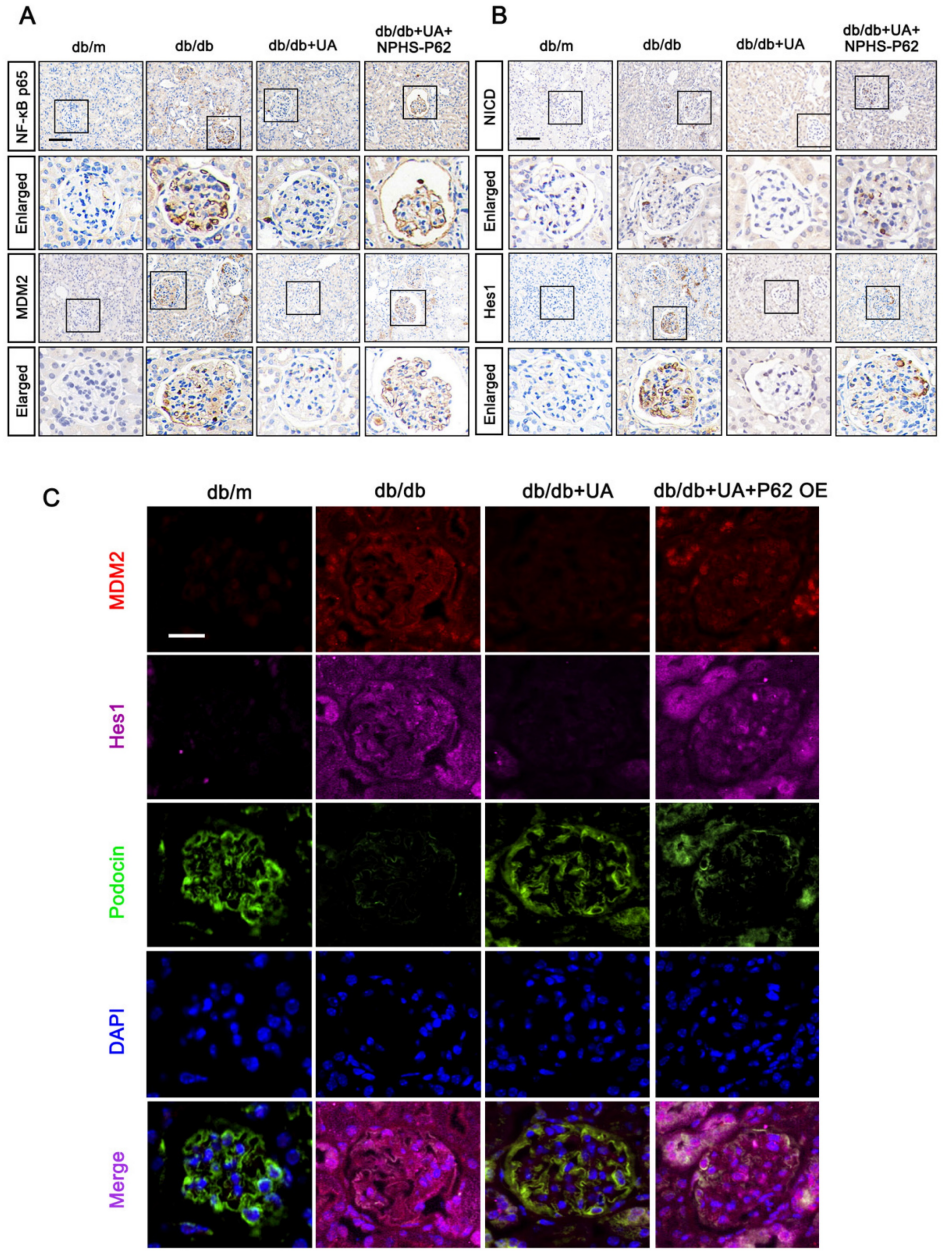
** UA protects podocyte from** MC** by inhibiting the P62-mediated NF-κB-MDM2-Notch1 pathway in DN progression.** (A) Protein levels of NF-κB p65, MDM2, NICD, and Hes1 in db/m, db/db, db/db + UA, and db/db + UA + NPHS-P62 groups by IHC assay (scale bar=50 μm); (B) Immunostained for MDM2 (red), Hes1 (pink), and Podocin (green), and counterstained with DAPI (blue) in db/m, db/db, db/db + UA, and db/db +UA + NPHS-P62 groups (scale bar=20 μm).

Furthermore, we identified the functions of the P62-NF-κB-MDM2 axis in the UA-treated db/db group. As shown in Figure 9A and 9B, the expression levels of NF-κB p65, MDM2, NICD, and Hes1 were down-regulated in the UA-treated db/db group, exhibiting levels nearly identical to the control group (db/m). However, NPHS-P62 significantly activated the NF-κB-MDM2 pathway, which was inhibited by UA administration. Additionally, similar trends were observed by the triple staining of MDM2 (Red), Podocin (Green), and Hes1 (Pink) in Figure 9C. Compared with the db/m group, an enhanced fluorescent intensity of MDM2 and Hes1 and decreased fluorescent intensity of Podocin were observed in the db/db group, and these were simultaneously reversed in the UA-treated db/db group. Over-expressed P62 enhanced the fluorescent intensity of MDM2 and Hes1 while reducing the expression of Podocin in the UA-treated db/db group, indicating that UA prevented podocyte MC through the P62-mediated MDM2-Notch1 pathway.

## 4. Discussion

Our research unveiled a novel mechanism of UA in mitigating podocyte dysfunction against DN progression. We proposed that in diabetic conditions, autophagic P62 accumulation regulates the expression of NF-κB and MDM2, and activates the downstream Notch1 signaling pathway. This abnormal activation of the Notch1 pathway causes podocytes to re-enter the cell cycle by suppressing G2/M phase arrest, leading to MC. However, UA administration can inhibit P62 accumulation and the P62-regulated expression of MDM2, thus correcting aberrant cell-cycle regulation by inhibiting Notch1 activation and delaying the prognosis of DN.

DN often begins with proteinuria, an early sign of renal injury that precipitates further progressive kidney destruction 29. As the final filtration barrier of the glomerulus, the onset of proteinuria results from the loss of podocytes 30. Numerous studies have indicated that defective autophagic flux in podocytes plays a crucial role in DN 12, 31, 32. Initially, lower fluorescent intensity of LC3 was observed, accompanied by abnormal nuclear shapes. Thus, we hypothesized that defective autophagy is responsible for podocyte MC, resulting in the down-regulation of podocyte cytoskeletal proteins, while the catastrophe is evidenced by the loss of cell-cycle control 33, 34. Previous studies have shown that P62 function depends on NRF2-driven MDM2 induction and p53-dependent and/or independent activity of MDM2 35. In the current research, autophagy substrate degradation inhibitors, such as Baf A1, further exacerbated podocyte MC, confirming that P62 is involved in mediating this catastrophe through autophagy.

This is supported by the correlation between impaired autophagic flux and aggravated podocyte injuries both i*n vitro* (HG culture) and *in vivo* (STZ-induced or db/db models) 12, 13, 36. Similar to our findings, UA-induced autophagy in oral squamous cell carcinoma cells (OSCC), as evidenced by LC3B-II conversion and increased P62 expression and accumulation of autophagosomes 31. We demonstrated that P62-mediated autophagic flux plays a crucial role in regulating DM-induced podocyte activities and the recovery of damaged podocytes under UA treatment. P62, known as the first-identified selective autophagy substrate, is generally considered to be inversely proportional to autophagic activity 32. Prior studies have shown that podocytes with siP62 effectively reduce podocyte injury induced by either high glucose or autophagic inhibitors 33. Our data indicate that the protective function of UA on podocytes under diabetic conditions is attributed to an increased level of overall autophagy. However, P62 accumulation, induced by using Baf A1, disrupts the normal autophagy flux, resulting in a diminished effect of UA administration, which may be a primary mechanism of UA function.

In addition to its role as an autophagy receptor recognizing poly-ubiquitinated proteins and organelles, P62 can interact directly or indirectly with a variety of signals involved in regulating cell survival and death, inflammation, and metabolic reprogramming 25. Previously studies have shown that autophagy inhibition leads to the accumulation of P62/SQSTM1 and activation of NF-κB, wherein NF-κB inhibition or P62/SQSTM1 knockdown attenuates PD-L1 induction by autophagy inhibition, revealing that autophagy regulates PD-L1 expression in gastric cancer through the P62/SQSTM1-NF-κB pathway 34. Additionally, activation of lung fibroblast CD148 reduces P62 accumulation, exerting antifibrotic effects by inhibiting NF-κB-mediated pro-fibrotic gene expression 35. These studies collectively suggest that autophagic P62 aggregation can activate the NF-κB signaling pathway, which is implicated in the pathogenesis of various diseases. Given that MDM2 serves as a co-transcription factor for the NF-κB pathway, NF-κB can promote the expression of the oncogene MDM2 36, 37. Furthermore, the tea polyphenol EGCG synergistically enhances the effects of DOX in inhibiting bladder cancer cell proliferation and migration via the NF-κB/MDM2/p53 pathway 38. MDM2 levels are suppressed with siP62, yet up-regulated in the presence of siP62 and TNF-α under HG conditions. The pronounced therapeutic effect of UA is diminished by over-expressed P62 or TNF-α administration, suggesting that UA alleviates podocyte injuries through the P62-NF-κB-MDM2 axis in DN conditions.

The Notch1 pathway, crucial for cell differentiation during organism development 39, 40, becomes aberrantly activated in mature kidneys, contributing significantly to the progression of proteinuria due to glomerular injury and podocyte lesions 27, 28. Previous research has shown that MDM2 activates the Notch signaling pathway through non-degradative ubiquitination and synergizes with Notch1 to inhibit apoptosis and promote proliferation 41, 42. Similarly, UA administration can down-regulate the expression of MDM2, accompanied by reduced levels of NICD and Hes1, indicating that the MDM2-regulated Notch1 signaling pathway is involved in the treatment of DN with UA, both *in vitro* and *in vivo*.

Considering that the MDM2/Notch1 signal is a crucial part of cell cycle regulation in podocytes, the upcoming issue to address is the correlation between the MC of podocytes and the MDM2-Notch1 pathway activated by P62 in DN progression. Generally, apart from apoptosis, which is a main reason for the loss of podocytes in DN, MC is reported to occur during DN development 23, 43. Differentiated podocytes face inherent obstacles to mitosis; their proliferative response does not promote recovery from injury but rather leads to the formation of multiple nuclei with irregular shapes and abnormal mitotic spindles that contribute to cytokinesis failure and aneuploidy 8, 23, 44. Mature podocytes exit from the cell cycle, as evidenced by lower expression of proliferating markers such as Ki67, PCNA, and Cyclin B1. Consistent with these findings, our data show that podocytes under HG conditions re-enter the cell cycle by upregulating levels of Ki67, Cyclin A, Cyclin B1, and P21, progressing to the S, G2/M, and M phases. These abnormal cell cycle events are due to the activation of the P62-mediated MDM2-Notch1 signaling under diabetic conditions, helping mature podocytes overcome the G2/M checkpoint. The accumulation of P62 increases MDM2-Notch1 activities, leading to glomerular disorders in DN. However, as discussed above, UA administration can regulate P62-mediated autophagy flux and sequentially inhibit the abnormal activation of the MDM2-Notch1 pathway, preventing podocyte death during the initial phases of glomerular injury and thus alleviating glomerulosclerosis. Nonetheless, the targeting of UA on podocytes and the low oral absorption and bioavailability of UA remain challenges to be overcome in clinical therapy, which will be comprehensive addressed in future studies.

## Conclusions

In the present study, we provided the mechanism of UA's protective effect during DN progression. UA administration can stabilize the cell cycle of podocytes, avoid MC, and alleviate podocyte injuries under diabetic conditions by inhibiting the abnormal activation of the autophagic P62-mediated NF-κB p65-MDM2-Notch1 signaling pathway.

## Supplementary Material

Supplementary figures.

## Abbreviations

AAV: Adeno-associated virus
ANOVA: One-way analysis of variance
Baf A1: Bafilomycin A1
BUN: Blood urine nitrogen
Cr: Creatinine
DAPI: 4',6-diamidino-2-phenylindole
DM: Diabetes mellitus
DN: Diabetic nephropathy
ELISA: Enzyme-linked immunosorbent assay
ESRD: End-stage renal disease
FN: Fibronectin
GBM: Glomerular basement membrane
GFB: Glomerular filtration barrier
Hes1: Hairy and enhancer of split 1
HG: High glucose
IDF: International Diabetes Federation
IHC: Immunohistochemical
IF: Immunofluorescent
LC3I/II: Microtubule-Associated Protein 1 Light Chain 3 I/II
MC: Mitotic catastrophe
MDM2: Murine Double Minute gene 2
NF-κB: Nuclear factor-κ-gene binding
NG: Normal glucose
NICD: Notch intracellular domain
Notch1: Neurogenic locus notch homolog protein 1
PAS: Periodic acid-Schiff
PCNA: proliferating cell nuclear antigen
p-H3: phospho-Histone H3
PI: Propidium Iodide
Rapa: Rapamycin
siRNA: Small interfering RNA
SPF: Specific pathogen-free
Synap: Synaptopodin
TEM: Transmission electron microscopy
TNF-α: Tumor necrosis factor-α
UA: Ursolic acid
UACR: Urine albumin/creatinine ratio

## References

[1] Magliano DJ, Boyko EJ, committee IDFDAtes (2021). IDF Diabetes Atlas. Idf diabetes atlas. Brussels: International Diabetes Federation© International Diabetes Federation, 2021.

[2] Umanath K, Lewis JB (2018). Update on Diabetic Nephropathy: Core Curriculum 2018. Am J Kidney Dis.

[3] Kriz W, Lowen J, Grone HJ (2023). The complex pathology of diabetic nephropathy in humans. Nephrol Dial Transplant.

[4] Naaman SC, Bakris GL (2023). Diabetic Nephropathy: Update on Pillars of Therapy Slowing Progression. Diabetes Care.

[5] Bronstein R, Pace J, Gowthaman Y, Salant DJ, Mallipattu SK (2023). Podocyte-Parietal Epithelial Cell Interdependence in Glomerular Development and Disease. J Am Soc Nephrol.

[6] Langham RG, Kelly DJ, Cox AJ, Thomson NM, Holthöfer H, Zaoui P (2002). Proteinuria and the expression of the podocyte slit diaphragm protein, nephrin, in diabetic nephropathy: effects of angiotensin converting enzyme inhibition. Diabetologia.

[7] Mulay SR, Thomasova D, Ryu M, Kulkarni OP, Migliorini A, Bruns H (2013). Podocyte loss involves MDM2-driven mitotic catastrophe. J Pathol.

[8] Zhan P, Zhang Y, Shi W, Liu X, Qiao Z, Wang Z (2022). Myeloid-derived growth factor deficiency exacerbates mitotic catastrophe of podocytes in glomerular disease. Kidney Int.

[9] Saito R, Rocanin-Arjo A, You YH, Darshi M, Van Espen B, Miyamoto S (2016). Systems biology analysis reveals role of MDM2 in diabetic nephropathy. JCI Insight.

[10] Yamamoto H, Zhang S, Mizushima N (2023). Autophagy genes in biology and disease. Nature reviews Genetics.

[11] Liu WJ, Ye L, Huang WF, Guo LJ, Xu ZG, Wu HL (2016). p62 links the autophagy pathway and the ubiqutin-proteasome system upon ubiquitinated protein degradation. Cell Mol Biol Lett.

[12] Barutta F, Bellini S, Kimura S, Hase K, Corbetta B, Corbelli A (2023). Protective effect of the tunneling nanotube-TNFAIP2/M-sec system on podocyte autophagy in diabetic nephropathy. Autophagy.

[13] Xu L, Fan Q, Wang X, Li L, Lu X, Yue Y (2017). Ursolic acid improves podocyte injury caused by high glucose. Nephrol Dial Transplant.

[14] López-Hortas L, Pérez-Larrán P, González-Muñoz MJ, Falqué E, Domínguez H (2018). Recent developments on the extraction and application of ursolic acid. A review. Food research international (Ottawa, Ont).

[15] Tasneem S, Liu B, Li B, Choudhary MI, Wang W (2019). Molecular pharmacology of inflammation: Medicinal plants as anti-inflammatory agents. Pharmacol Res.

[16] Yin R, Li T, Tian JX, Xi P, Liu RH (2018). Ursolic acid, a potential anticancer compound for breast cancer therapy. Critical reviews in food science and nutrition.

[17] Silva FS, Oliveira PJ, Duarte MF (2016). Oleanolic, Ursolic, and Betulinic Acids as Food Supplements or Pharmaceutical Agents for Type 2 Diabetes: Promise or Illusion?. Journal of agricultural and food chemistry.

[18] Ma TK, Xu L, Lu LX, Cao X, Li X, Li LL (2019). Ursolic Acid Treatment Alleviates Diabetic Kidney Injury By Regulating The ARAP1/AT1R Signaling Pathway. Diabetes Metab Syndr Obes.

[19] Ma T, Li X, Zhu Y, Yu S, Liu T, Zhang X (2022). Excessive Activation of Notch Signaling in Macrophages Promote Kidney Inflammation, Fibrosis, and Necroptosis. Front Immunol.

[20] Guo J, Zheng W, Liu Y, Zhou M, Shi Y, Lei M (2023). Long non-coding RNA DLX6-AS1 is the key mediator of glomerular podocyte injury and albuminuria in diabetic nephropathy by targeting the miR-346/GSK-3β signaling pathway. Cell Death Dis.

[21] Mitrofanova A, Fontanella A, Tolerico M, Mallela S, Molina David J, Zuo Y (2022). Activation of Stimulator of IFN Genes (STING) Causes Proteinuria and Contributes to Glomerular Diseases. J Am Soc Nephrol.

[22] Tang H, Lei CT, Ye C, Gao P, Wan C, Chen S (2017). MDM2 is implicated in high-glucose-induced podocyte mitotic catastrophe via Notch1 signalling. J Cell Mol Med.

[23] Wang Z, Chang Y, Liu Y, Liu B, Zhen J, Li X (2022). Inhibition of the lncRNA MIAT prevents podocyte injury and mitotic catastrophe in diabetic nephropathy. Mol Ther Nucleic Acids.

[24] Tao M, Liu T, You Q, Jiang Z (2020). p62 as a therapeutic target for tumor. European journal of medicinal chemistry.

[25] Thomasova D, Mulay SR, Bruns H, Anders HJ (2012). p53-independent roles of MDM2 in NF-κB signaling: implications for cancer therapy, wound healing, and autoimmune diseases. Neoplasia (New York, NY).

[26] Thomasova D, Bruns HA, Kretschmer V, Ebrahim M, Romoli S, Liapis H (2015). Murine Double Minute-2 Prevents p53-Overactivation-Related Cell Death (Podoptosis) of Podocytes. J Am Soc Nephrol.

[27] Li MR, Lei CT, Tang H, Yin XJ, Hao Z, Qiu Y (2022). MAD2B promotes podocyte injury through regulating Numb-dependent Notch 1 pathway in diabetic nephropathy. Int J Biol Sci.

[28] Wang XM, Yao M, Liu SX, Hao J, Liu QJ, Gao F (2014). Interplay between the Notch and PI3K/Akt pathways in high glucose-induced podocyte apoptosis. Am J Physiol Renal Physiol.

[29] Oshima M, Shimizu M, Yamanouchi M, Toyama T, Hara A, Furuichi K (2021). Trajectories of kidney function in diabetes: a clinicopathological update. Nature reviews Nephrology.

[30] Jefferson JA, Shankland SJ, Pichler RH (2008). Proteinuria in diabetic kidney disease: a mechanistic viewpoint. Kidney Int.

[31] Lin CW, Chin HK, Lee SL, Chiu CF, Chung JG, Lin ZY (2019). Ursolic acid induces apoptosis and autophagy in oral cancer cells. Environ Toxicol.

[32] Fan X, Huang T, Tong Y, Fan Z, Yang Z, Yang D (2022). p62 works as a hub modulation in the ageing process. Ageing research reviews.

[33] Wang B, Qian JY, Tang TT, Lin LL, Yu N, Guo HL (2021). VDR/Atg3 Axis Regulates Slit Diaphragm to Tight Junction Transition via p62-Mediated Autophagy Pathway in Diabetic Nephropathy. Diabetes.

[34] Wang X, Wu WKK, Gao J, Li Z, Dong B, Lin X (2019). Autophagy inhibition enhances PD-L1 expression in gastric cancer. J Exp Clin Cancer Res.

[35] Tsoyi K, Liang X, De Rossi G, Ryter SW, Xiong K, Chu SG (2021). CD148 Deficiency in Fibroblasts Promotes the Development of Pulmonary Fibrosis. Am J Respir Crit Care Med.

[36] Hu Y, Jin R, Gao M, Xu H, Zou S, Li X (2019). Transcriptional repression of IKKβ by p53 in arsenite-induced GADD45α accumulation and apoptosis. Oncogene.

[37] Zhu D, Zheng S, Fang C, Guo X, Han D, Tang M (2020). Dysbindin promotes pancreatic ductal adenocarcinoma metastasis by activating NF-κB/MDM2 via miR-342-3p. Cancer Lett.

[38] Luo KW, Zhu XH, Zhao T, Zhong J, Gao HC, Luo XL (2020). EGCG Enhanced the Anti-tumor Effect of Doxorubicine in Bladder Cancer via NF-κB/MDM2/p53 Pathway. Front Cell Dev Biol.

[39] Dietrich B, Haider S, Meinhardt G, Pollheimer J, Knöfler M (2022). WNT and NOTCH signaling in human trophoblast development and differentiation. Cell Mol Life Sci.

[40] Matsumoto K, Luther KB, Haltiwanger RS (2021). Diseases related to Notch glycosylation. Mol Aspects Med.

[41] Kim H, Ronai ZA (2018). Rewired Notch/p53 by Numb'ing Mdm2. The Journal of cell biology.

[42] Singh S, Vaughan CA, Rabender C, Mikkelsen R, Deb S, Palit Deb S (2019). DNA replication in progenitor cells and epithelial regeneration after lung injury requires the oncoprotein MDM2. JCI Insight.

[43] Hara M, Oohara K, Dai DF, Liapis H (2019). Mitotic Catastrophe Causes Podocyte Loss in the Urine of Human Diabetics. Am J Pathol.

[44] Altintas MM, Reiser J (2019). Podocytes: Way to Go. Am J Pathol.

