# Supplementary material for: Ursolic Acid Alleviates Mitotic Catastrophe in Podocyte by Inhibiting Autophagic P62 Accumulation in Diabetic Nephropathy
Source: Int J Biol Sci. 2024 Jun 11;20(9):3317–33. doi: 10.7150/ijbs.94096 (PMC11234211; doi:10.7150/ijbs.94096)
Supplement: Supplementary file 1 — Supplementary figures. [file ijbsv20p3317s1.pdf]

Supplementary Materials

Ursolic Acid Alleviates Mitotic Catastrophe in Podocyte by Inhibiting Autophagic P62 Accumulation in Diabetic Nephropathy

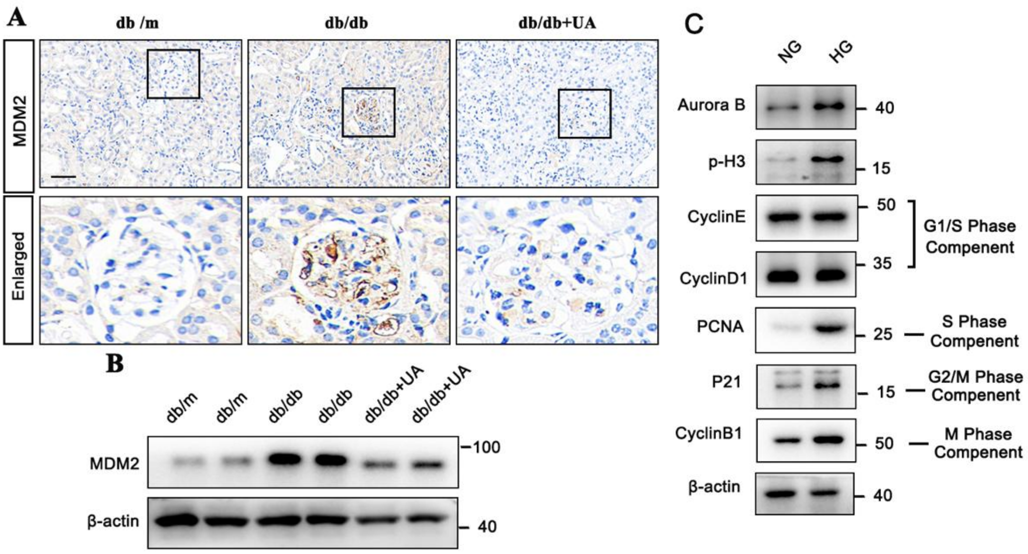

Figure S1. HG induces podocyte MC with up-regulation of MDM2. (A) Protein level of MDM2 in db/m, db/db, and db/db + UA groups by IHC assay (scale bar=50 μm); (B) Protein level of MDM2 in db/m, db/db, and db/db + UA groups by western blot (n=6); (C) Protein levels of Aurora B, p-H3, Cyclin E, Cyclin D1, PCNA, P21 and Cyclin B1 in NG and HG groups by western blot (n=3).

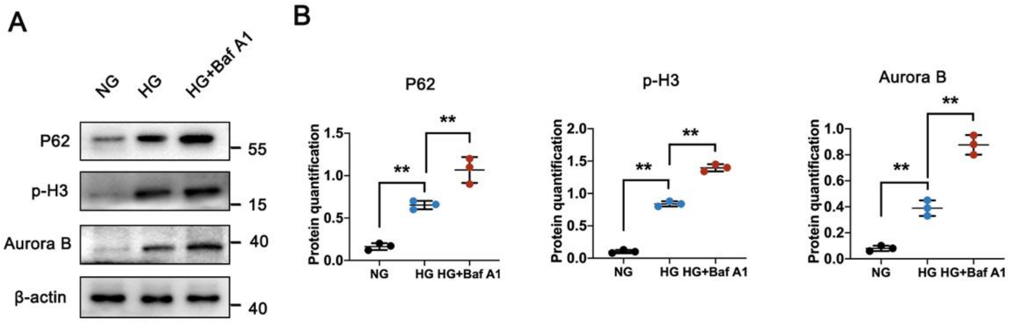

Figure S2. Defective autophagy leads to the podocyte MC induced by HG. (A, B) Protein levels of P62, p-H3, and Aurora B in NG, HG, and HG + Baf A1 groups by western blot, with semi-quantitative analyses (n=3). Data represented the mean ± SD of three independent experiments. **P<.01 or ***P<.001 versus HG group by one-way ANOVA.

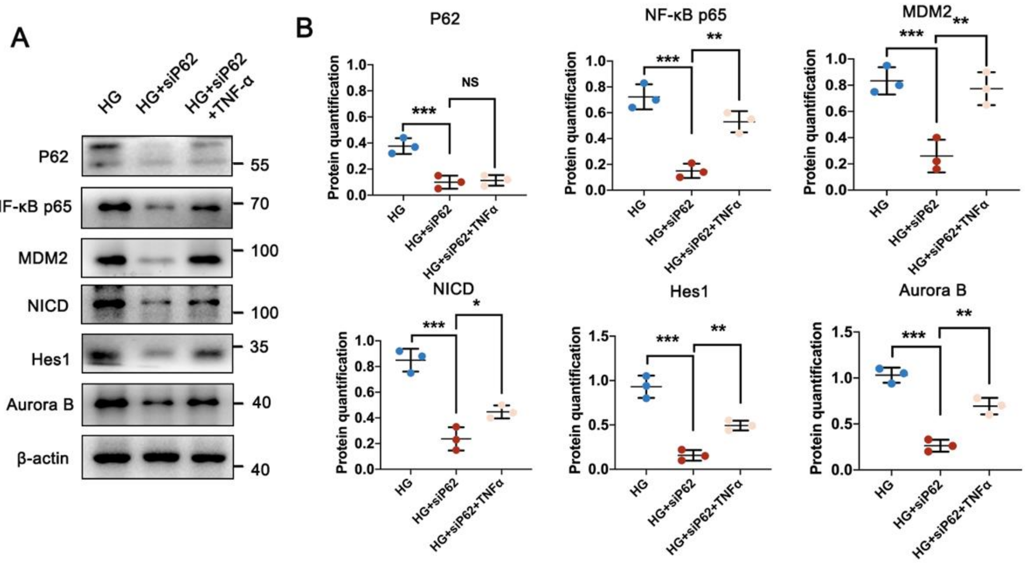

Figure S3. P62-mediated NF-κB/MDM2 pathway induces podocyte MC. (A, B) Protein levels of P62, NF-κB p65, MDM2, NICD, Hes1 and Aurora B in HG, HG + siP62, and HG + siP62 + TNF-α groups by western blot, with semi-quantitative analyses (n=3). Data represented the mean ± SD of three independent experiments. **P<.01 or ***P<.001 versus HG + siP62 group by one-way ANOVA.
